# Tetra­aqua­bis(3-carboxyl­atopyridine *N*-oxide-κ*O* ^3^)cadmium(II)

**DOI:** 10.1107/S1600536809022430

**Published:** 2009-06-17

**Authors:** Chao-Yan Zhang, Qian Gao, Yue Cui, Ya-Bo Xie

**Affiliations:** a College of Environmental and Energy Engineering, Beijing University of Technology, Beijing 100022, People’s Republic of China

## Abstract

In the title complex, [Cd(C_6_H_4_NO_3_)_2_(H_2_O)_4_], the Cd^II^ atom is situated on a crystallographic centre of inversion. The Cd^II^ atom shows a slightly distorted octa­hedral geometry and is coordinated by four O atoms from water mol­ecules and two O atoms from deprotonated carboxyl groups of nicotinic acid *N*-oxide ligands. The mononuclear complex mol­ecules are linked by O—H⋯O hydrogen bonds, forming a three-dimensional network structure.

## Related literature

For a related stucture, see: Hilkka *et al.* (1983 ▶).
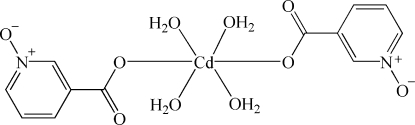


## Experimental

### 

#### Crystal data

[Cd(C_6_H_4_NO_3_)_2_(H_2_O)_4_]
*M* *_r_* = 460.67
Monoclinic, 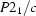

*a* = 8.896 (2) Å
*b* = 13.284 (3) Å
*c* = 6.902 (1) Å
β = 106.95 (3)°
*V* = 780.2 (3) Å^3^
*Z* = 2
Mo *K*α radiation
μ = 1.46 mm^−1^
*T* = 293 K
0.24 × 0.24 × 0.24 mm

#### Data collection

Bruker SMART CCD area-detector diffractometer
Absorption correction: multi-scan (*SADABS*; Bruker, 1998 ▶) *T*_min_ = 0.705, *T*_max_ = 0.712
3886 measured reflections
1371 independent reflections
1216 reflections with *I* > 2σ(*I*)
*R*_int_ = 0.013

#### Refinement

*R*[*F* ^2^ > 2σ(*F* ^2^)] = 0.017
*wR*(*F* ^2^) = 0.047
*S* = 1.11
1371 reflections
115 parameters
H-atom parameters constrained
Δρ_max_ = 0.26 e Å^−3^
Δρ_min_ = −0.27 e Å^−3^

### 

Data collection: *SMART* (Bruker, 1998 ▶); cell refinement: *SAINT* (Bruker, 1998 ▶); data reduction: *SAINT*; program(s) used to solve structure: *SHELXS97* (Sheldrick, 2008 ▶); program(s) used to refine structure: *SHELXL97* (Sheldrick, 2008 ▶); molecular graphics: *SHELXTL* (Sheldrick, 2008 ▶); software used to prepare material for publication: *SHELXTL*.

## Supplementary Material

Crystal structure: contains datablocks I, global. DOI: 10.1107/S1600536809022430/im2121sup1.cif

Structure factors: contains datablocks I. DOI: 10.1107/S1600536809022430/im2121Isup2.hkl

Additional supplementary materials:  crystallographic information; 3D view; checkCIF report

## Figures and Tables

**Table 1:** Hydrogen-bond geometry (Å, °)

*D*—H⋯*A*	*D*—H	H⋯*A*	*D*⋯*A*	*D*—H⋯*A*
O1*W*—H1*WA*⋯O2^i^	0.85	1.90	2.678 (2)	151
O1*W*—H1*WB*⋯O3^ii^	0.85	1.86	2.697 (2)	165
O2*W*—H2*WA*⋯O3^iii^	0.86	1.86	2.716 (2)	175
O2*W*—H2*WB*⋯O2^ii^	0.86	1.93	2.787 (2)	173

Symmetry codes: (i) 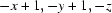; (ii) 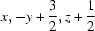; (iii) 
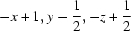
.

**Fig. 1.**
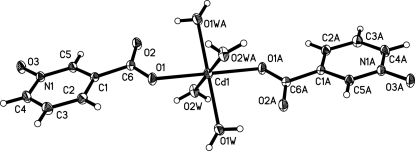
The molecular structure of the title compound with displacement ellipsoids drawn at the 30% probability level for non-hydrogen atoms. Symmetry related atoms labelled A have the symmetry code A = -x + 1, -y + 1, -z.

**Fig. 2.**
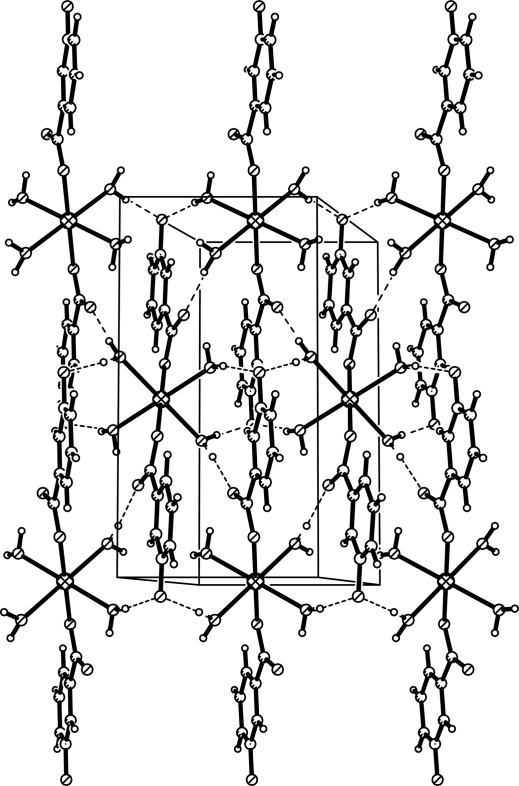
Supramolecular structure of the title compound realized by O—H···O hydrogen bond.

## supplementary crystallographic information

### Comment

The behaviour of nicotinic acid N-oxide ligand towards transition metals has been studied (Hilkka *et al.*, 1983). Herein, we prepared a new complex with the similar structure.

The title complex (Fig. 1) is made up of tetraaquametal cations and nicotinate N-oxide anion. The Cd^II^ centre shows a slightly distorted octahedral geometry and is six-coordinated by four O atoms from water molecules and two O atoms from deprotonated carboxylic groups of nicotinic acid N-oxide ligands. The O atoms of the N-oxide function bridge two water ligands of adjacent complex molecules *via* O—H···O hydrogen bonds, forming infinite chains along *c* axis (Fig. 2). Otherwise, the chains are linked by additional O—H···O hydrogen bonds observed between carboxyl O atoms and H atoms of coordinated water molecules. In conclusion, the mononucear complexes are linked by O—H···O hydrogen bonds, forming a three-dimensional network structure.

### Experimental

A solution containing a 1 : 1 : 2 molar ratio of nicotinic acid N-oxide, LiOH × H_2_O and Cd(NO_3_)_2_ × 4 H_2_O in water was sealed in a 25 ml teflon reactor and kept at 140° for 3 days. The mixture was stepwise cooled to 40° with a rate of 10° per hour and was then allowed to cool to room temperature naturally. Colorless block-shaped crystals suitable for X-ray investagation were collected from the final mixture.

### Refinement

All H atoms were fixed geometrically (C—H = 0.93 Å, O—H = 0.85–0.86 Å) and treated as riding with *U*_iso_(H) = 1.2*U*_eq_(carrier).

### Crystal data

**Table:**

[Cd(C_6_H_4_NO_3_)_2_(H_2_O)_4_]	*F*(000) = 460
*M**_r_* = 460.67	*D*_x_ = 1.961 Mg m^−^^3^
Monoclinic, *P*2_1_/*c*	Mo *K*α radiation, λ = 0.71073 Å
Hall symbol: -P 2ybc	Cell parameters from 2694 reflections
*a* = 8.896 (2) Å	θ = 2.4–30.8°
*b* = 13.284 (3) Å	µ = 1.46 mm^−^^1^
*c* = 6.902 (1) Å	*T* = 293 K
β = 106.95 (3)°	Block, colorless
*V* = 780.2 (3) Å^3^	0.24 × 0.24 × 0.24 mm
*Z* = 2	

### Data collection

**Table:**

Bruker SMART CCD area-detector diffractometer	1371 independent reflections
Radiation source: fine-focus sealed tube	1216 reflections with *I* > 2σ(*I*)
graphite	*R*_int_ = 0.013
φ and ω scans	θ_max_ = 25.0°, θ_min_ = 2.4°
Absorption correction: multi-scan (*SADABS*; Bruker, 1998)	*h* = −10→9
*T*_min_ = 0.705, *T*_max_ = 0.712	*k* = −15→13
3886 measured reflections	*l* = −8→8

### Refinement

**Table:**

Refinement on *F*^2^	Primary atom site location: structure-invariant direct methods
Least-squares matrix: full	Secondary atom site location: difference Fourier map
*R*[*F*^2^ > 2σ(*F*^2^)] = 0.017	Hydrogen site location: inferred from neighbouring sites
*wR*(*F*^2^) = 0.047	H-atom parameters constrained
*S* = 1.11	*w* = 1/[σ^2^(*F*_o_^2^) + (0.0239*P*)^2^ + 0.3039*P*] where *P* = (*F*_o_^2^ + 2*F*_c_^2^)/3
1371 reflections	(Δ/σ)_max_ = 0.001
115 parameters	Δρ_max_ = 0.26 e Å^−^^3^
0 restraints	Δρ_min_ = −0.27 e Å^−^^3^

### Special details

**Table:**

Geometry. All e.s.d.'s (except the e.s.d. in the dihedral angle between two l.s. planes) are estimated using the full covariance matrix. The cell e.s.d.'s are taken into account individually in the estimation of e.s.d.'s in distances, angles and torsion angles; correlations between e.s.d.'s in cell parameters are only used when they are defined by crystal symmetry. An approximate (isotropic) treatment of cell e.s.d.'s is used for estimating e.s.d.'s involving l.s. planes.
Refinement. Refinement of *F*^2^ against ALL reflections. The weighted *R*-factor *wR* and goodness of fit *S* are based on *F*^2^, conventional *R*-factors *R* are based on *F*, with *F* set to zero for negative *F*^2^. The threshold expression of *F*^2^ > σ(*F*^2^) is used only for calculating *R*-factors(gt) *etc*. and is not relevant to the choice of reflections for refinement. *R*-factors based on *F*^2^ are statistically about twice as large as those based on *F*, and *R*- factors based on ALL data will be even larger.

### Fractional atomic coordinates and isotropic or equivalent isotropic displacement parameters (Å^2^)

**Table:**

	*x*	*y*	*z*	*U*_iso_*/*U*_eq_	
Cd1	0.5000	0.5000	0.0000	0.02694 (9)	
O1	0.69494 (17)	0.61434 (10)	0.0646 (2)	0.0322 (3)	
O2	0.54243 (17)	0.73900 (11)	−0.1003 (2)	0.0356 (4)	
O3	0.84106 (18)	1.04656 (11)	0.1475 (2)	0.0351 (4)	
C5	0.7630 (2)	0.88034 (15)	0.0896 (3)	0.0258 (4)	
H5A	0.6623	0.9017	0.0195	0.031*	
C1	0.7954 (2)	0.77885 (14)	0.1108 (3)	0.0234 (4)	
O1W	0.6079 (2)	0.42109 (12)	0.3026 (2)	0.0475 (5)	
C6	0.6669 (2)	0.70560 (15)	0.0175 (3)	0.0257 (4)	
C4	1.0221 (2)	0.92024 (16)	0.2703 (3)	0.0323 (5)	
H4A	1.0988	0.9685	0.3232	0.039*	
O2W	0.37122 (19)	0.60651 (11)	0.1620 (2)	0.0387 (4)	
N1	0.8750 (2)	0.94858 (13)	0.1691 (3)	0.0263 (4)	
C2	0.9455 (3)	0.74878 (15)	0.2168 (3)	0.0293 (5)	
H2A	0.9699	0.6807	0.2349	0.035*	
C3	1.0585 (2)	0.82017 (17)	0.2953 (3)	0.0349 (5)	
H3A	1.1600	0.8004	0.3656	0.042*	
H1WA	0.5831	0.3597	0.2735	0.042*	
H1WB	0.6909	0.4234	0.4033	0.042*	
H2WA	0.3009	0.5861	0.2151	0.042*	
H2WB	0.4182	0.6543	0.2406	0.042*	

### Atomic displacement parameters (Å^2^)

**Table:**

	*U*^11^	*U*^22^	*U*^33^	*U*^12^	*U*^13^	*U*^23^
Cd1	0.02508 (13)	0.02246 (14)	0.02930 (14)	−0.00329 (8)	0.00169 (9)	−0.00103 (8)
O1	0.0296 (8)	0.0189 (7)	0.0431 (9)	−0.0038 (6)	0.0024 (7)	0.0023 (6)
O2	0.0315 (8)	0.0226 (8)	0.0428 (9)	−0.0029 (6)	−0.0046 (7)	0.0027 (7)
O3	0.0339 (8)	0.0176 (7)	0.0483 (9)	−0.0020 (6)	0.0034 (7)	−0.0013 (7)
C5	0.0213 (10)	0.0232 (10)	0.0301 (11)	−0.0017 (8)	0.0031 (8)	−0.0001 (8)
C1	0.0270 (10)	0.0196 (10)	0.0235 (10)	−0.0022 (8)	0.0072 (8)	−0.0001 (8)
O1W	0.0593 (11)	0.0263 (8)	0.0391 (9)	−0.0105 (8)	−0.0133 (8)	0.0035 (7)
C6	0.0279 (11)	0.0219 (11)	0.0269 (10)	−0.0035 (8)	0.0073 (9)	−0.0012 (8)
C4	0.0241 (11)	0.0301 (12)	0.0378 (12)	−0.0073 (9)	0.0015 (9)	−0.0026 (9)
O2W	0.0365 (9)	0.0331 (8)	0.0477 (10)	−0.0077 (7)	0.0143 (7)	−0.0101 (7)
N1	0.0269 (9)	0.0200 (9)	0.0300 (9)	−0.0021 (7)	0.0052 (7)	−0.0007 (7)
C2	0.0314 (11)	0.0217 (11)	0.0326 (12)	0.0014 (8)	0.0061 (9)	0.0013 (9)
C3	0.0239 (11)	0.0328 (12)	0.0420 (13)	0.0008 (9)	0.0006 (9)	0.0025 (10)

### Geometric parameters (Å, °)

**Table:**

Cd1—O1^i^	2.2499 (14)	C1—C2	1.382 (3)
Cd1—O1	2.2499 (14)	C1—C6	1.496 (3)
Cd1—O1W^i^	2.2836 (16)	O1W—H1WA	0.8537
Cd1—O1W	2.2836 (16)	O1W—H1WB	0.8538
Cd1—O2W	2.3045 (16)	C4—N1	1.345 (3)
Cd1—O2W^i^	2.3045 (16)	C4—C3	1.367 (3)
O1—C6	1.261 (2)	C4—H4A	0.9300
O2—C6	1.248 (2)	O2W—H2WA	0.8559
O3—N1	1.335 (2)	O2W—H2WB	0.8603
C5—N1	1.340 (3)	C2—C3	1.373 (3)
C5—C1	1.377 (3)	C2—H2A	0.9300
C5—H5A	0.9300	C3—H3A	0.9300
			
O1^i^—Cd1—O1	180.0	Cd1—O1W—H1WA	102.3
O1^i^—Cd1—O1W^i^	91.98 (6)	Cd1—O1W—H1WB	139.5
O1—Cd1—O1W^i^	88.02 (6)	H1WA—O1W—H1WB	109.2
O1^i^—Cd1—O1W	88.02 (6)	O2—C6—O1	125.51 (19)
O1—Cd1—O1W	91.98 (6)	O2—C6—C1	118.05 (17)
O1W^i^—Cd1—O1W	180.00 (7)	O1—C6—C1	116.44 (18)
O1^i^—Cd1—O2W	92.70 (6)	N1—C4—C3	119.74 (19)
O1—Cd1—O2W	87.30 (6)	N1—C4—H4A	120.1
O1W^i^—Cd1—O2W	91.49 (7)	C3—C4—H4A	120.1
O1W—Cd1—O2W	88.51 (7)	Cd1—O2W—H2WA	122.8
O1^i^—Cd1—O2W^i^	87.30 (6)	Cd1—O2W—H2WB	122.7
O1—Cd1—O2W^i^	92.70 (6)	H2WA—O2W—H2WB	104.2
O1W^i^—Cd1—O2W^i^	88.51 (7)	O3—N1—C5	119.82 (16)
O1W—Cd1—O2W^i^	91.49 (7)	O3—N1—C4	119.00 (16)
O2W—Cd1—O2W^i^	180.0	C5—N1—C4	121.18 (18)
C6—O1—Cd1	120.98 (13)	C3—C2—C1	119.49 (19)
N1—C5—C1	120.73 (18)	C3—C2—H2A	120.3
N1—C5—H5A	119.6	C1—C2—H2A	120.3
C1—C5—H5A	119.6	C4—C3—C2	120.2 (2)
C5—C1—C2	118.64 (18)	C4—C3—H3A	119.9
C5—C1—C6	118.74 (18)	C2—C3—H3A	119.9
C2—C1—C6	122.62 (18)		
			
O1^i^—Cd1—O1—C6	178 (100)	C5—C1—C6—O1	−170.21 (19)
O1W^i^—Cd1—O1—C6	39.82 (16)	C2—C1—C6—O1	10.0 (3)
O1W—Cd1—O1—C6	−140.18 (16)	C1—C5—N1—O3	−179.78 (18)
O2W—Cd1—O1—C6	−51.77 (16)	C1—C5—N1—C4	0.4 (3)
O2W^i^—Cd1—O1—C6	128.23 (16)	C3—C4—N1—O3	179.46 (19)
N1—C5—C1—C2	0.4 (3)	C3—C4—N1—C5	−0.7 (3)
N1—C5—C1—C6	−179.34 (18)	C5—C1—C2—C3	−1.0 (3)
Cd1—O1—C6—O2	−15.1 (3)	C6—C1—C2—C3	178.8 (2)
Cd1—O1—C6—C1	165.45 (13)	N1—C4—C3—C2	0.2 (4)
C5—C1—C6—O2	10.3 (3)	C1—C2—C3—C4	0.7 (3)
C2—C1—C6—O2	−169.5 (2)		

Symmetry codes: (i) −*x*+1, −*y*+1, −*z*.

### Hydrogen-bond geometry (Å, °)

**Table:**

*D*—H···*A*	*D*—H	H···*A*	*D*···*A*	*D*—H···*A*
O1W—H1WA···O2^i^	0.85	1.90	2.678 (2)	151
O1W—H1WB···O3^ii^	0.85	1.86	2.697 (2)	165
O2W—H2WA···O3^iii^	0.86	1.86	2.716 (2)	175
O2W—H2WB···O2^ii^	0.86	1.93	2.787 (2)	173

Symmetry codes: (i) −*x*+1, −*y*+1, −*z*; (ii) *x*, −*y*+3/2, *z*+1/2; (iii) −*x*+1, *y*−1/2, −*z*+1/2.
